# Vowst’s FDA approval is a boon for the prevention of recurrent *Clostridioides difficile* infection

**DOI:** 10.1097/MS9.0000000000001410

**Published:** 2023-10-17

**Authors:** Ayush Anand, Nameera Parveen Shaikh, Yash Aggarwal, Umaima Fatima, Sanskriti Chapagain, Rahul Chidurala, Jenish Vaghela, Arihant Surana, Charmy Parikh, Raj H. Patel

**Affiliations:** a B.P. Koirala Institute of Health Sciences, Dharan; b Devdaha Medical College and Research Institute, Rupandehi, Nepal; c Government Institute of Medical Sciences, Greater Noida; d Shadan Institute of Medical Sciences, Hyderabad, Telangana; e B.J. Medical College, Ahmedabad, Gujarat; f Sri Ramachandra Institute of Higher Education and Research, Chennai, Tamil Nadu, India; g Batumi Shota Rustaveli State University, Batumi, Georgia; h Department of Internal Medicine, Saint Vincent Hospital, Worcester, Massachusetts; i Department of Internal Medicine, Carle BroMenn Medical Center, Normal, Illinois; j Department of Internal Medicine, St. Mary Medical Center, Pennsylvania, USA; k Global Consortium of Medical Education and Research, Pune, India

*Dear Editor,*

*Clostridioides difficile* (*C. difficile*) is an anaerobic, Gram-positive, spore-forming bacteria frequently found in the intestines of both humans and animals, leading to *Clostridioides difficile* infection (CDI)^1,2^. The global prevalence of *C. difficile* in food samples ranges from 4.8 to 8.2%, with the highest prevalence in seafood^3^. The clinical picture of signs and symptoms vary from asymptomatic to severe fulminant colitis, toxic megacolon, intestinal perforation, and multiple organ dysfunction syndrome^1,2^. The diagnosis is mainly based on clinical presentation and laboratory investigations to detect *C. difficile* and toxins in feces^4^.

Various treatment and preventive options such as actoxumab, bezlotoxumab, surotomycin, cadazolid, and Cdiffense have been used for reducing CDI burden. Despite global efforts to decrease the burden in the past decade, recent studies have reported an increasing global burden of CDI, with a median incidence of ~4 per 10 000 patient days^5^. Approximately 10–20% of the CDI cases are due to recurrent CDI (rCDI), defined as an episode of CDI occurring within 8 weeks following the earlier episode^5,6^. Factors such as advancing age, antibiotic use, malignancy, heart disease, leukocyte count, length of hospital stay, intensive care unit admission, and antiulcer medications are significantly related to rCDI^5,7,8^. In the USA, rCDI is a significant public health challenge, accounting for ~75 000–175 000 cases annually^5,7^. In addition to the economic burden on patients, an active episode of rCDI significantly impairs the quality of life of these patients^9^. All these mandates for effective prevention strategies to decrease the incidence of rCDI.

So far, the treatment of rCDI has been focused mainly on using antimicrobials against the vegetative form of *C. difficile*. However, on 26 April 2023, the U.S. Food and Drug Administration (FDA) approved Vowst (SER-109), the first orally administered fecal microbiota transplantation (FMT), to prevent adult rCDI^10^. Vowst is a formulation of live fecal microbiota consisting of Firmicutes spores. This formulation paved the way for another approach to prevent rCDI by using FMT to address the microbiota disruption caused by CDI. Though the exact mechanism of action of Vowst has not yet been established, there are a few ways through which it can act. Firstly, when the healthy bacteria from the donors’ gut interacts directly with the *C. difficile*, the donors’ gut bacteria can compete for resources with *C. difficile* and may produce bacteriocins to stop the growth or kill *C. difficile* directly^11,12^. Also, FMT increases the levels of beneficial bacteria such as *Bacteroidetes*, *Clostridium* clusters IV and XIVa, *Fecalibacterium prausnitzii*, *Butyrivibrio crossotus*, Enterococcus, *Lactobacillus*, and *Veillonella* ^12,13^. At the same time, it reduces harmful bacteria like *C. difficile*, *Enterococcus aerogenes*, and *Caudovirales* ^12–14^. Another important factor contributing to successful management is the concentration of viruses in FMT. Those who responded well to FMT had more donor *Caudovirales* in their gut^15^. Also, increased donor-derived *Caudovirales* in the recipients’ gut leads to a significantly higher cure rate after FMT^15^. Hence, considering virome composition in the donor selection can improve FMT effectiveness.

The phase 2 trial of Vowst did not show a significant difference in rCDI in the SER-109 and placebo groups^16^. However, a significant reduction in rCDI was reported in people aged over 65 years in the SER-109 group^16^. Also, early engraftment of SER-10 was significantly associated with reduced rCDI and increased concentration of secondary bile acids^16^. In addition, adverse events were usually mild to moderate^16^. The FDA approval of Vowst, formerly known as SER-109, was based on promising results from ECOSPOR III, a double-blind, placebo-controlled, randomized trial^17^. In this trial, after antimicrobial therapy with vancomycin or fidaxomicin, the rCDI patients received either oral SER-109 (four capsules thrice daily for 3 days on an empty stomach) or placebo. The study revealed a significantly reduced risk of rCDI in the SER-109 group (Relative Risk = 0.32; 95% CI = 0.18–0.58) than the placebo group at 8 weeks. The efficacy of SER-109 in reducing rCDI was significantly higher at 4 weeks, 8 weeks, 12 weeks, and 24 weeks^18^. Also, a reduced incidence of rCDI was noted irrespective of age group and specific antibiotic treatment. Furthermore, the engraftment of spore-forming Firmicutes bacteria was higher in the SER-109 group, leading to sustained clinical response. This engraftment is essential as it helps form secondary bile acids, which are bacteriostatic for *C difficile*. In addition to the sustained clinical response of SER-109 in preventing rCDI, no serious adverse effects possibly or directly related to SER-109 were reported. However, adverse events such as gastrointestinal disorders (flatulence, abdominal distension, abdominal pain, constipation, diarrhea, nausea, vomiting), fatigue, chills, and decreased appetite were reported in the majority of the participants in both SER-109 and placebo groups. The adverse events were mild to moderate. Though deaths were reported in the SER-109 group, none could be linked to SER-109 usage. Overall, the ECOSPOR III trial showed that SER-109 could be used safely and effectively in reducing rCDI.

Another ECOSPOR IV trial assessed the recurrence rate of CDI and found that SER-109 leads to a significantly reduced risk of rCDI, irrespective of age, number of prior recurrences, and diagnostic approach^19^. Similar to the ECOSPOR III trial, the ECOSPOR IV trial also reported gastrointestinal side effects, which were mild to moderate and resolved independently. Though ~12.5% of patients developed severe adverse events and mortality in 3.0% of patients, none were attributed to SER-109 use. Despite the proven safety and efficacy of SER-109, equal importance should be given to understanding the quality of life in these patients. A secondary analysis of the ECOSPOR III trial reported a significant improvement in health-related quality of life at 1 week, with continued improvement at week 8^20,21^. This shows the substantial impact of SER-109 treatment on the quality of life in rCDI patients. In addition, the oral administration route of SER-109 makes it more patient-compliant and tolerable and gives a significant unparalleled advantage over other FMTs.

Though the clinical application and associated beneficial impact on quality of life in rCDI is critical, cost-effectiveness is an equally vital trait to demonstrate the feasibility of any intervention approach^22^. The current studies have not assessed the cost-effectiveness of using Vowst on a large scale. Hence, further studies are warranted to assess the cost-effectiveness of Vowst. However, a study was conducted to assess the impact of SER-109 on healthcare resource utilization (HRU), defined as hospitalizations or emergency visits due to rCDI^23^. This study found a significantly lower cumulative incidence of HRU at 4 weeks (5.62 vs. 19.35%, *P*-value =0.004) and 8 weeks (11.24 vs. 22.58%, *P*-value =0.020)^23^. Since HRU reflects an indirect measure of the healthcare burden, we can conclude that Vowst can help reduce the healthcare burden due to rCDI.

Overall, safety, efficacy, and reduction in healthcare burden make Vowst an ideal candidate for implication as an rCDI prevention. However, further studies are warranted to investigate its effects on pregnant females, lactating mothers, oncological patients, immunosuppressive patients, and comorbid patients with indications for other antimicrobials and malabsorption syndrome.

## Ethical approval

Ethical approval is not applicable for this Letter to Editor.

## Consent

Informed consent is not applicable for this Letter to Editor

## Sources of funding

The authors did not receive any funding for this work.

## Author contribution

A.A.: conceptualization, supervision, writing – original draft, review, and editing; N.P.S., Y.A., U.F., S.C., and R.C.: writing – original draft, review, and editing; J.V.: conceptualization, writing – original draft, review, and editing; A.S., C.P., and R.H.P.: supervision and writing – review and editing.

## Conflicts of interest disclosure

The authors do not have any conflicts of interest to declare.

## Research registration unique identifying number (UIN)

Not applicable.

## Guarantor

Ayush Anand.

## Data availability statement

Not applicable.

## Provenance and peer review

Not commissioned, externally peer-reviewed.
